# Marine Sulfated Polysaccharides: Preventive and Therapeutic Effects on Metabolic Syndrome: A Review

**DOI:** 10.3390/md19110608

**Published:** 2021-10-27

**Authors:** Ying Li, Juan Qin, Yinghui Cheng, Dong Lv, Meng Li, Yanxia Qi, Jing Lan, Qiancheng Zhao, Zhibo Li

**Affiliations:** 1 College of Food Science and Engineering, Dalian Ocean University, Dalian 116023, China; liying@dlou.edu.cn (Y.L.); qinjuan202107@163.com (J.Q.); yinghuicheng2021@163.com (Y.C.); donglv2019@163.com (D.L.); limeng@dlou.edu.cn (M.L.); qiyanxia@dlou.edu.cn (Y.Q.); 2 Dalian Key Laboratory of Marine Bioactive Substances Development and High Value Utilization, Dalian 116023, China; 3 Collaborative Innovation Center of Seafood Deep Processing, Dalian Polytechnic University, Dalian 116034, China; 4 Liaoning Provincial Aquatic Products Analyzing, Testing and Processing Technology Scientific Service Centre, Dalian 116023, China; 5 Key Laboratory of Aquatic Product Processing and Utilization of Liaoning Province, Dalian 116023, China; 6 Dalian Zhenjiu Biological Industry Co., Ltd., Dalian 116023, China; fly06161011@163.com

**Keywords:** marine sulfate polysaccharides, metabolic syndrome, gut microbiota, functional foods, sulfate

## Abstract

Metabolic syndrome is the pathological basis of cardiovascular and cerebrovascular diseases and type 2 diabetes. With the prevalence of modern lifestyles, the incidence of metabolic syndrome has risen rapidly. In recent years, marine sulfate polysaccharides (MSPs) have shown positive effects in the prevention and treatment of metabolic syndrome, and they mainly come from seaweeds and marine animals. MSPs are rich in sulfate and have stronger biological activity compared with terrestrial polysaccharides. MSPs can alleviate metabolic syndrome by regulating glucose metabolism and lipid metabolism. In addition, MSPs prevent and treat metabolic syndrome by interacting with gut microbiota. MSPs can be degraded by gut microbes to produce metabolites such as short chain fatty acids (SCFAs) and free sulfate and affect the composition of gut microbiota. The difference between MSPs and other polysaccharides lies in the sulfation pattern and sulfate content, therefore, which is very important for anti-metabolic syndrome activity of MSPs. This review summarizes the latest findings on effects of MSPs on metabolic syndrome, mechanisms of MSPs in treatment/prevention of metabolic syndrome, interactions between MSPs and gut microbiota, and the role of sulfate group and sulfation pattern in MSPs activity. However, more clinical trials are needed to confirm the potential preventive and therapeutic effects on human body. It may be a better choice to develop new functional foods containing MSPs for dietary intervention in metabolic syndrome.

## 1. Introduction

**Figure 1:**
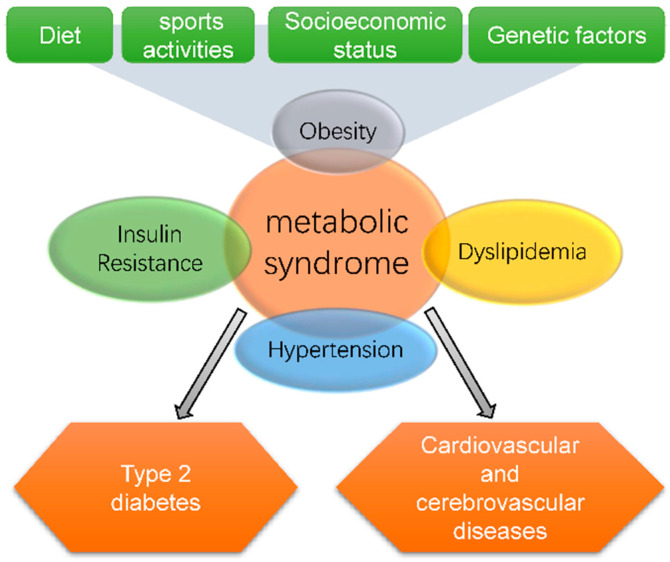
Metabolic syndrome is interconnected with type 2 diabetes and cardiovascular and cerebrovascular diseases.

Cardiovascular diseases have become the leading killer of human beings [1]. Many risk factors contribute to cardiovascular disease, such as genetics, aging, smoking, and metabolic syndrome [2]. Genetics and aging cannot be modified, and metabolic syndrome can be addressed by lifestyle modifications or pharmacologic treatment. Metabolic syndrome mainly includes obesity, insulin resistance, atherogenic dyslipidemia, and hypertension [3]. Modern diets and lifestyles, such as high intake of sugar, fat and salt, and less exercise have led to an increasing incidence of metabolic syndrome (Figure 1) [4]. Dietary change is the main strategy for the treatment of metabolic syndrome, and the development of functional food components is an important method. In recent years, marine sulfate polysaccharides (MSPs) have shown to be effective in the prevention and treatment of metabolic syndrome [5,6].

Sulfated polysaccharides (SPs) are sulfated derivatives of polysaccharides, which are multifunctional active compounds formed by substituting some hydroxyl groups of monosaccharide molecules in the chain of polysaccharides by sulfate radical [7]. Due to the special environment of the sea, polysaccharides from marine organisms tend to contain more sulfate groups than polysaccharides from terrestrial microorganisms, animals, and plants [8,9]. A variety of MSPs have been isolated from seaweed and marine animals, such as fucosylated chondroitin sulfate, chondroitin sulfate, fucoidan, keratan sulfate, heparin sulfate, carrageenans, and ulvans [10,11]. The diverse biological activities of MSPs, including antiobesity, anticoagulant, immunomodulating, antihyperlipidemic and antioxidative, have attracted remarkable interest. Among various functions, the anti-metabolic syndrome function of MSPs was only discovered in recent years [6,12,13,14]. Studies have shown that MSPs can treat metabolic syndrome by regulating the dysbiosis of gut microbiota and the production of related metabolites [15]. Therefore, elucidating the effects of MSPs on gut microbiota, especially the mechanism of MSPs interaction with gut microbiota, will help to explain the mechanism of MSPs against metabolic syndrome and further promote their application.

MSPs cannot be digested directly by the human body but can only be absorbed and utilized through the fermentation of gut microorganisms [16]. Gut microbiota is a general term for the microbial community living in the human gut. It is one of the most attractive research focuses in the fields of microbiology, medicine, and genetics in recent years [17,18]. Many scientists refer to the gut microbiota as the body’s organ due to its large number and complex interaction with the human body [19]. Gut microbiota is closely related to human health, which can help digestion, regulate immunity, avoid pathogen invasion, regulate mood, etc. Evidence has shown that gut microbiota dysbiosis is related to obesity, diabetes, cardiovascular diseases, and cancer [20]. The interaction between MSPs and gut microbiota plays an important role in metabolic syndrome. The establishment of relevant dietary intervention strategies has become a hot research topic in the field of functional food.

More than 30,000 research papers from 2000 to 2021 were found in PubMed by searching keywords such as sulfate polysaccharides, metabolic syndrome, gut microbiota, obesity, insulin resistance, atherogenic dyslipidemia, and hypertension, etc., and effects of MSPs on metabolic syndrome, regulation of MSPs on gut microbiota, utilization of MSPs in gut microbiota, mechanism of MSPs in treatment/prevention of metabolic syndrome, and therapeutic methods and dietary intervention.

## 2. Effects of MSPs on Metabolic Syndrome

The metabolic syndrome mainly includes obesity, insulin resistance, atherogenic dyslipidemia, and hypertension [3]. Representative studies on the effects of MSPs on the metabolic syndrome are listed in Table 1.

### 2.1. Obesity

Obesity is a state of pathological and physiological changes caused by excessive accumulation of fat in the human body. Obesity not only affects physical beauty, but also brings inconvenience to life. In addition, it can also cause a variety of diseases, including cardiovascular diseases, joint soft tissue damage, psychological disorders, diabetes, fatty liver, etc. [36]. Drug treatment for obesity, so far, mainly involves blocking the absorption of food or increasing metabolism. Drug use may cause side effects and drug resistance, and drug treatment must be carefully selected. Therefore, food-derived natural products with obesity prevention and treatment functions are of interest to researchers, and among them, MSPs are more promising. MSPs from *Cymodocea nodosa* effectively reduced weight and inhibited lipase activity in obese mice [21], and MSPs from brown seaweed *Ascophyllum nodosum* could pass through the upper digestive tract and could be degraded by gut microbe, thus regulating the composition of gut microbiota and reducing the risk of obesity [22]. MSPs of abalone gonad could improve the metabolic syndrome and gut microbiota dysbiosis induced by high fat diet and could affect nutrient absorption and energy metabolism [23]. SP from sea cucumber has a good therapeutic effect on diet-induced obesity mice; it could reduce the levels of fat, serum lipid and inflammatory cytokines, and had a good preventive effect on metabolic syndrome [24]. A variety of MSPs from different sources showed positive effects in the prevention and treatment of obesity, and they seem to be potential ingredients for the development of new drugs and dietary treatment of obesity. However, current research only stays in the animal experiment stage, and further clinical trials are needed to verify their role and safety in human obesity.

### 2.2. Insulin Resistance

The decrease in the efficiency with which insulin promotes glucose uptake and utilization is known as insulin resistance. It is one of important pathogenesis of cardiovascular diseases and type 2 diabetes [37]. Insulin resistance promotes cardiovascular disease by increasing the vascular stiffness. Elevated plasma insulin level, along with a concomitant decrease in bioavailable nitric oxide, plays an important role in the pathogenesis of impaired vascular relaxation and vascular stiffness in patients with cardiovascular disease [38]. Fucoidan, a favorable MSP, has a promising therapeutic effect on insulin resistance [39,40]. Fucoidan exists in a variety of marine organisms and is mainly composed of L-fucose and sulfate groups. However, the structure of fucoidan varies according to its source [5]. Fucoidan from *Sargassum fusiforme* improved insulin resistance by activating the Nrf2 pathway, regulating the structure of gut microbiota, and reducing intestinal inflammation [25]. However, fucoidan extract from *Fucus vesiculosus* showed no marked effect on insulin resistance in obese, nondiabetic subjects [26]. This might be due to the different structure of fucoidan from different sources. In addition to fucoidan, fucosylated chondroitin sulfate-dominated polysaccharide fraction from *Cucumaria frondosa* improved insulin resistance by activating IRS/PI3K/AKT signaling and regulating GSK-3β gene expression in T2DM rats [27]. In summary, various MSPs from different sources have therapeutic potential against insulin resistance; however, the treatment of insulin resistance might have to use an appropriate and specific MSP.

### 2.3. Dyslipidemia

Dyslipidemia is an abnormal metabolism of lipoprotein in human body, and it is one of important factors leading to atherosclerosis and an independent risk factor for coronary heart disease and ischemic stroke [41]. Studies have shown that MSPs have hypolipidemic effects. Fucoidan is effective in reducing dyslidemia and atherosclerosis, inducing lipoprotein lipase activity, and suppressing inflammatory and oxidative stress in mice [28]. The administration of MSP from *Sargassum vulgare* to obese rats could inhibit lipase activity, regulate lipid profile, and limit lipid peroxidation [29]. The sulfate of MSPs may have a significant effect on the hypolipidemic effect. MSPs from sea cucumbers *Pearsonothuria graeffei* dominated with a 4-O-sulfation pattern had a significant effect on lipid regulation; however, MSPs from sea cucumbers *Isostichopus badionotus* dominated with a 2-O-sulfation pattern showed limited effects [30]. In another work, Ulvan PU3 from *Ulva pertusa* with the highest uronic acid and sulfate content exhibited stronger antioxidant activities than other ulvans in the model of hyperlipidemic Kunming mice [31]. Therefore, the sulfation pattern and sulfate content in MSPs might contribute to the regulation of dyslipidemia.

### 2.4. Hypertension

Hypertension is the most common chronic disease and the most important risk factor for cardiovascular and cerebrovascular diseases. Because high blood pressure requires lifelong treatment, safe and reliable medications are important. Low molecular weight fucoidan seems to have a good effect on hypertension, and studies showed that it could improve basic hypertension in type 1 and type 2 diabetic rats [32,33]. In rat experiments, fucoidan from *Undaria Pinnatifida* was found to effectively and consistently reduce high blood pressure, even after the drug was stopped, indicating its excellent potential in the treatment of hypertension [34]. Carrageenan might also have potential for the treatment of hypertension, as its producer *Sarconema filiforme* was effective in improving diet-induced metabolic syndrome. Systolic blood pressure was significantly reduced in rats with diet-induced metabolic syndrome after 5% *S. filiforme* supplementation [35].

## 3. Mechanism of MSPs in Treatment/Prevention of Metabolic Syndrome

**Figure 2:**
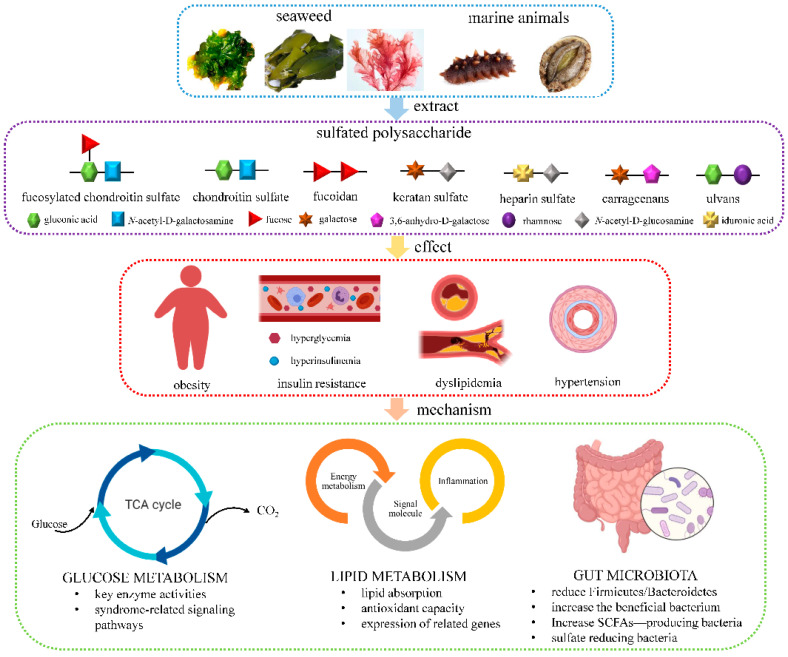
Scope for MSPs involved in source, characteristics, effect on health and its mechanism. This figure was created with icons provided by bio render (https://biorender.com; accessed on 22 October 2021). Abbreviations: TCA, tricarboxylic acid; SCFAs, short chain fatty acids.

MSPs treat or prevent metabolic syndrome through regulating glucose or lipid metabolism based on microbiota-targeting strategies. The scope for MSPs involved in source, characteristics, effect on health, and its mechanism was shown in Figure 2. Metabolites such as dihydroxyacetone phosphate, acetyl coenzyme A, and NADPH are generated during glucose metabolism, which provide precursors for lipid metabolism. However, when cells are blocked from using glucose, the body uses fat oxidation for energy and generate acetyl coenzyme A, thus entering the tricarboxylic acid cycle and affecting glucose metabolism. Therefore, the regulation of glucose and lipid metabolism by MSPs may be a cyclic pathway.

### 3.1. Regulating Glucose Metabolism

Although a number of studies have shown that MSPs have significant effects in the treatment of metabolic syndrome, it should be noted that most of the studies focus on the function of MSPs, and the research on the mechanism is still in progress, which is related to the complex mechanism of MSPs in the treatment of metabolic syndrome. Some studies focus on the effects of MSPs on the key enzyme activities related to metabolic syndrome, for example, fucoidan is an inhibitor of α glucosidase, which can reduce postprandial hyperglycemia [42]. High molecular weight fucoidan from *F. vesiculosus* can also indirectly reduce postprandial hyperglycemia through the inhibition of dipeptidyl peptidase-IV (DPP-IV) [43]. DPP-IV is an enzyme that is involved in the inhibition of the rapid degradation of incretin hormones, which prevents postprandial hyperglycemia. Inhibiting DPP-IV prolongs the action of incretins, which reduces glucose production and increases insulin production [44]. Additionally, some studies have examined changes in metabolic syndrome-related signaling pathways and gene expression following MSPs intake. The sulfated rhamnose polysaccharides from *Enteromorpha prolifera* have significant effects on glycemic control by activating the insulin receptor/insulin receptor substrate-2/phosphoinositide 3-kinase/protein kinase B/glycogen synthase kinase 3β (IR/IRS-2/PI3K/PKB/GSK-3β) signaling pathway, which is related to glycogen synthesis, to improve glucose metabolism and enhance glucose transport through glucose transporter 4 translocation [45]. MSPs from sea cucumber also improve glucose metabolism by activating the insulin-mediated PI3K/PKB/GSK-3β signaling pathway, improving insulin resistance and promoting glycogen accumulation [27,46]. Low molecular weight fucoidan activates adenosine monophosphate-activated protein kinase to prevent metabolic syndrome by controlling endoplasmic reticulum stress-dependent pathways [47]. Repairing islet cells to increase insulin secretion and increasing glycogen synthesis to increase glucose utilization are beneficial to reducing blood glucose content. The MSPs from *U. pinnatifida* have a strong α-glucosidase inhibitory activity, which can improve glucose intake, reduce islet cell damage, and promote liver glycogen synthesis [48].

### 3.2. Regulating Lipid Metabolism

A number of experimental studies have shown that most MSPs are effective in lipid metabolism. MSPs could regulate lipid metabolism by affecting lipid absorption and improving antioxidant capacity. Goto et al. found that sacran, an MSP mainly from *Aphanothece sacrum*, could reduce the increase in serum triglyceride induced by corn oil in SD rats and had the potential of reducing lipids absorption into blood [49]. It could also reduce the level of oxidative stress in serum and hepatic injury, accompanied by increased levels of transforming growth factor-β and tumor necrosis factor-α. Chondroitin sulfate from salmon nasal cartilage inhibited fat storage in mice fed a high-fat diet, possibly by inhibiting pancreatic lipase activity and fatty acid uptake through the brush border membrane, thus inhibiting the absorption of dietary fat in the small intestine [50]. MSP from *Cystoseira crinita* could inhibit lipase activity in plasma and small intestine, leading to a significant decrease in blood triglyceride in rats fed a high fat diet [51]. In mice induced by high fat diet, it was found that polysaccharides from abalone viscera not only decreased blood lipid but also significantly increased the activity of malondialdehyde and superoxide dismutase, suggesting that it might play a role in the antioxidant pathway to improve the lipid metabolism disorder [52]. Ethanol extract from *Hypnea musciformis* could modulate lipid peroxidation in mammary carcinogenesis [53]. MSPs also affect lipid metabolism by regulating the expression of related genes. Reportedly, 200 mg kg^−1^ MSP from *E. prolifera* could significantly reduce the expression of hydroxy methylglutaryl coenzyme A (HMG-CoA) reductase and sterol regulatory element binding protein-2 (SREBP-2) in the liver of non-alcoholic fatty liver disease rats induced by a high-fat diet, in which SREBP-2 is a key transcription factor of cholesterol metabolism, while inhibiting HMG-CoA reductase could hinder cholesterol synthesis [54]. Fucoidan has been extensively studied on the mechanism of lipid regulation. It mainly transferred lipids from blood to liver and promoted lipid metabolism by regulating the expression of genes related to reverse cholesterol transport and lipid metabolism in hyperlipidemic C57BL/6J mice, such as low-density lipoprotein receptor, scavenger receptor B type 1, cholesterol 7 alpha-hydroxylase A1, peroxisome proliferator-activated receptor (PPAR) α, liver X receptor β, ATP-binding cassette transporter A1, and sterol regulatory element-binding protein 1c [55]. Fucoidan also decreased the expression of PPARγ (peroxisome proliferator-activated receptor γ, major transcription factors associated with adipocyte differentiation) in the liver of apolipoprotein E-deficient mice fed a high-fat diet [56]. Another study showed that sea cucumber fucoidan significantly increased the expression of Wnt/β-catenin pathway related genes and decreased the expression of key transcription factors such as SREBP-1c, CCAAT/enhancer binding protein-α (C/EBPα) and PPARγ in the high-fat-high-fructose diet mice. β-catenin inhibited the expression of PPARγ and C/EBPα, thus slowing adipocyte differentiation, while SREBP-1c had the opposite effect [57].

## 4. Regulation of MSPs on Gut Microbiota

In recent years, the study of gut microbiota has aroused widespread interest and provided a new idea for the study of the mechanism of MSPs in the prevention and treatment of metabolic syndrome. The interaction between MSPs and gut microbiota has been found in many studies [16,58]. MSPs can be metabolized by gut microbiota and its metabolites in turn affected the gut microbiota. The gut microbiota plays an important role in the metabolic syndrome, which was confirmed in human and animal experiments [59,60]. The clinical characterization of metabolic syndrome is the proliferation of potentially harmful bacteria and the inhibition of beneficial ones. Therefore, MSPs regulation of metabolic syndrome begins with gut microbial degradation, acts on the dysbiosis of gut microbiota, and has far-reaching effects beyond the gut. Representative studies on the regulation of MSPs on gut microbiota are listed in Table 2.

Chondroitin sulfate/fucosylated chondroitin sulfate usually showed powerful effects on the gut microbiota in mice. Shang et al. found that chondroitin sulfate had a positive regulation effect on gut microbiota, which was gender dependent [61]. Chondroitin sulfate significantly increased the number of *Bacteroides acidifaciens*, which indirectly played its role in gut immune regulation and improved the stress-induced intestinal inflammation [70]. Fucosylated chondroitin sulfate from sea cucumbers generally had the function of alleviating metabolic syndrome and gut microbiota dysbiosis, which could reduce the ratio of Firmicutes to Bacteroidetes and increase the abundance of beneficial bacterium *Megamonas*, *Bacteroides*, *Fusobacterium*, *Parabacteroides*, *Prevotella*, *Faecalibacterium* [62,63]. The ratio of Firmicutes to Bacteroidetes is generally regarded as an important indicator of obesity, and obese patients generally have a higher ratio [36].

Studies found that fucoidan could improve the dysbiosis of gut microbiota by increasing the abundance of Lactobacillus and Ruminococcaceae [64]. Lactobacillus are traditional probiotics, which have a variety of functions, while arterial stiffness correlates negatively with the abundance of Ruminococcaceae [71]. Another study in mice with gut microbiota dysbiosis caused by antibiotics showed that fucoidan not only increased the abundance of Ruminococcaceae, but also increased the abundance of *Akkermansia*, which was consistently correlated with metabolic syndrome and considered as the next generation probiotic [72]. Fucoidan could also reduce the Firmicutes to Bacteroidetes ratio in obese mice induced by high fat diet [25]. In diabetic mouse models, *S*. *fusiforme* fucoidan could relieve diabetic symptoms by regulating gut microbiota, and increasing the abundance of benign bacteria, such as *Bacteroides*, *Faecalibacterium*, and *Blautia* [65,66].

Carrageenan also has the effect of regulating gut microbiota in the treatment of metabolic syndrome, but its efficacy and safety need to be further studied, which may be related to its degree of polymerization [35,67]. A number of studies have shown that carrageenan induces colitis and is related to the gut microbiota of the host [68,73]. Porphyran and ulvans also have some anti-metabolic syndrome function, but there are few studies on related mechanism and its effect on gut microbiota [69,74].

## 5. Utilization of MSPs by Gut Microbiota

**Figure 3:**
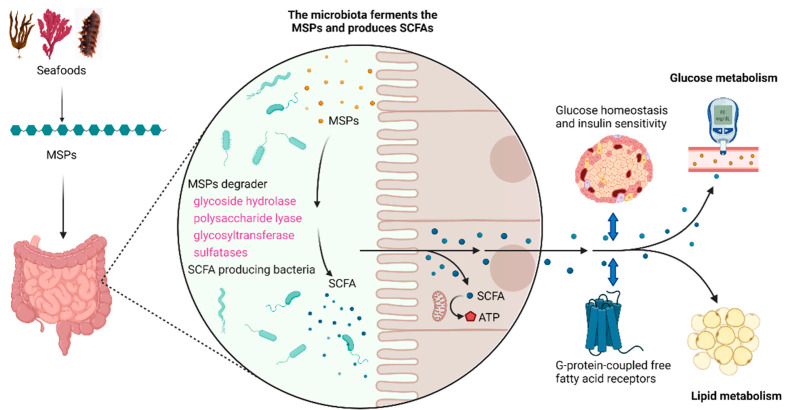
The utilization of MSPs by gut microbiota. This figure was created by bioRender (https://biorender.com; accessed on 22 October 2021). Abbreviations: SCFAs, short chain fatty acids; MSPs, marine sulfate polysaccharides; ATP, adenosine triphosphate.

Non-digestible polysaccharides are rarely digested by the human body itself but can be degraded by gut microbiota to produce short chain fatty acids (SCFAs) and other substances that can be absorbed by the human body. SCFAs can be used as an energy source of intestinal epithelial cells and have functions such as regulating gut microbiota dysbiosis and resistance to pathogen invasion [75]. The utilization of MSPs by gut microbiota was showed in Figure 3. Previous studies suggested that, unlike non-SPs, MSPs were poorly fermentable, which might be related to the degree of sulfation [76,77]. However, recent studies showed that small molecular weight MSPs (<30 kDa) could be degraded by human gut microbes and produce large amounts of SCFAs, which regulate the balance of gut microbiota [78]. MSPs from pacific abalone could be degraded by intestinal Bacteroidetes in an in vitro fermentation model, and it was species dependent [79]. These different results may have something to do with the fact that the gut microbiota of the Chinese, who traditionally consume kelp and other algae, is more suitable for the degradation of MSPs. MSPs from *A. nodosum* also showed strong in vitro digestibility and fermentability, and the total SCFA content increased significantly after the digestion [22]. Similar results could also be found in animal experiments. Feeding fucoidan from *Acaudina molpadioides* significantly increased the content of SCFAs and the abundance of SCFA producing bacteria *Coprococcus, Rikenella,* and *Butyricicoccus* in the gut of mice [80]. Therefore, it is of great significance to study the digestion of MSPs in gut to understand the mechanism of MSPs in the treatment of metabolic syndrome.

Gut microbes have a series of genes encoding the binding and subsequent degradation of MSPs, including genes encoding glycoside hydrolase, polysaccharide lyase, glycosyltransferase, and other related enzymes such as sulfatases [81]. These genes can be found in human gut microbes such as *Streptococcus*, *Eubacterium*, *Bifidobacterium*, *Faecalibacterium*, and *Bacteroides*, among which *Bacteroides* is the most important degrader [76], because it is dominant in the human gut, and genes mentioned above in *Bacteroides* form a tightly regulated colocalized gene clusters [82]. After degradation of MSPs, oligosaccharides, monosaccharides, SCFAs, and other metabolites are produced, some of which can be directly absorbed by the human body, while others can be used by other microbes through the cross-feeding mechanism.

SCFAs have a strong regulatory effect on gut microbiota, thus affecting the onset and development of metabolic syndrome. There are many SCFA-producing bacteria in gut microbiota, such as *Bacteroides*, *Alloprevotella*, *Clostridium*, *Eubacterium*, *Faecalibacterium*, *Roseburiam* and so on [83]. Different types of MSPs selectively increased the abundance of SCFA producing bacteria. For example, MSPs from sea cucumber significantly increased the abundance of *Bacteroides*, *Allobaculum*, *Alloprevotella*, *Roseburia*, and *Turicibacter* in BALB/c mice [84]. Nevertheless, fucosylated chondroitin sulfate from *A. molpadioides* increased the abundance of *Lactobacillus*, *Bifidobacterium*, and *Lachnospiraceae* NK4A136 group in obese mice [85]. SCFAs act as an active signaling molecule that interacts with G-protein-coupled free fatty acid receptors and has a wide range of effects on glucose and lipid metabolism [86]. SCFAs can improve glucose homeostasis and insulin sensitivity and directly reduce the secretion of adipose tissue-derived pro-inflammatory cytokines and chemokines [87]. More and more evidence shows that SCFA, as a metabolic tool, has a potential role in preventing and counteracting metabolic syndrome. Therefore, regulating SCFAs produced by the gut microbiota through the ingestion of MSPs is a useful tool for the prevention of metabolic syndrome.

Apart from the utilization of MSPs by gut microbiota, MSPs can be directly absorbed into the human body through the intestinal tract. There are many studies on the absorption and pharmacokinetics of MSPs by human body [88,89]. The absorption and pharmacokinetics of MSPs should be considered in correct doses and treatment schemes selection of metabolic syndrome. Studies have shown that high molecular weight fucoidans can be absorbed by rat intestinal epithelial cells but cannot accumulate in blood and urine [88]. MSPs can be detected in urine after ingestion in human bodies, and dietary habit is a factor in the absorption of MSPs [89]. The absorption rate of fucoidan via the intestine using subjects is 0.6% [90]. The pharmacokinetic and tissue distribution of fucoidan exhibit considerable heterogeneity. Fucoidan first accumulates in kidney (AUC_0–t_ = 10.74 μg·h/g; C_max_ = 1.23 μg/g after 5 h), spleen (AUC_0–t_ = 6.89 μg·h/g; C_max_ = 0.78 μg/g after 3 h), and liver (AUC_0–t_ = 3.26 μg·h/g; C_max_ = 0.53 μg/g after 2 h), and stays in the blood with a mean residence time (6.79 h) [91]. Studies on pharmacokinetics of fucoidan is useful for understanding the molecular mechanism of biological activity and correct selecting therapeutic doses.

## 6. Role of Sulfate Group and Sulphation Pattern

The function of MSPs is closely related to its sulphation patterns and sulfate group, and the sulfate group plays a special role in the interaction with gut microbiota [30,31,76]. The content of sulfate group is significantly different with different extraction methods and sources, which leads to significant differences in the activity of MSPs from different sources or even the same source. MSPs from abalone have a variety of fragments, and their activity is related to molecular weight and sulfate content [92,93]. A fraction named AGP33 with sulfates occur at 3-O- and 4-O-positions showed a better anticoagulant activity than its desulfated product [93]. MSPs from abalone viscera also exhibited significantly hypolipidemic and anti-atherogenic activities [52]. Another abalone SP (AGSP) and its desulfated product D-AGSP can inhibit fat accumulation and improve lipid metabolism balance and gut microbiota dysbiosis in mice fed with high fat diet [94]. However, there were significant differences in relative abundance of *Oscillibacter* and *Helicobacter* in gut microbiota between AGSP and D-AGSP groups, and the butyrate content in feces of mice in D-AGSP group was significantly lower than that in AGSP group, indicating that the sulfate group did not have a decisive effect on the hypolipidemic activity of AGSP, but it might affect the inhibitory effect on metabolic syndrome by regulating the composition of gut microbiota and the expression of their metabolites [94]. There are few studies relating to the effect of sulfate group in MSPs on metabolic syndrome, which might partly determine the activity of MSPs through gut microbiota; however, the specific mechanism is still unclear and needs further study.

After MSPs are degraded in the gut, they might generate free sulfate that is used by sulfate-reducing bacteria, such as *Desulfovibrio*, to form hydrogen sulfide [95]. Although hydrogen sulfide in the gut may protect the gut microbiota from reactive oxygen species, high concentration of hydrogen sulfide might be toxic to the host [95]. It seems important to control the abundance of sulfate-reducing bacteria as well as H_2_S levels in the intestines of people on high MSPs diet. However, whether sulfate-reducing bacteria is beneficial or harmful is still debated, and its role in the pathogenesis of metabolic diseases such as obesity remains unclear [96]. Therefore, tracking the free sulfate content of MSPs after degradation in gut to clarify the release of sulfate in MSPs and studying the physiological functions of sulfate reducing bacteria by colonizing germ-free mice is of great importance so as to understand the mechanism of sulfate group determining the activity of MSPs.

## 7. Therapeutic Methods and Dietary Intervention

A number of studies indicate that most MSPs have anti-metabolic syndrome effects, and most of them are based on animal experiments. However, few clinical trials have been carried out on fucoidan as an anti-metabolic syndrome drug (Table 3). Previous studies showed that 5 weeks administration of 400 mg fucoidan could effectively shorten lysis time of the thrombus [97], and three months 500 mg of fucoidan daily taking for obese and overweight people could lower diastolic blood pressure and low-density lipoprotein cholesterol and raise insulin levels [98]. The combination of fucoidan and fucoxanthin can improve hepatic steatosis, inflammation, fibrosis, and insulin resistance in non-alcoholic fatty liver disease patients [99]. However, a recent randomized controlled trial of obese patients showed no significant differences in insulin resistance or other measures of cardiometabolic health between the two groups given 90 days 500 mg of fucoidan and placebo [26]. In a clinical trial of carrageenan, carrageenan failed to reduce glycosylated hemoglobin and homeostasis model assessment-insulin resistance in prediabetic patients, while a diet without carrageenan improved insulin signaling and glucose tolerance [100]. Therefore, MSPs still need further research and development as anti-metabolic syndrome drugs. However, fucoidan, as an MSP entering clinical studies, is promising for its safety and effectiveness in managing metabolic syndrome.

Recently, there has been great interest in the appropriate use of MSPs to achieve maximum health benefits [7], and they seem to have important application in dietary intervention of metabolic syndrome as functional foods. For metabolic syndrome such as obesity and dyslipidemia, scientific and reasonable diet intervention combined with exercise is the safest and most effective basic treatment at present. However, the traditional low-calorie meal replacement mode is a disguised diet, which is not only uncomfortable and has poor compliance, but also easily causes a decline in immunity, stress gastritis, and other problems. Adding MSPs to a normal or even high-fat diet can prevent or alleviate metabolic syndrome [6,15,94], and this not only ensures the supply of energy and nutrition, but also achieves the purpose of reducing fat, blood glucose, and insulin resistance. It is especially suitable for people who are easy to regain weight and is not suitable for exercise, obese and overweight children, pregnant women, and people with type 2 diabetes. In addition, direct consumption of MSPs-rich seafood also has some remission effect on metabolic syndrome. Red seaweed *S. filiforme* and *Kappaphycus alvarezii*, the producers of carrageenan, can be used as functional foods to relieve metabolic syndrome [35,101]. Many brown seaweeds also have good effects on metabolic syndrome management, owing to high amounts of MSPs in the total dry mass [102]. Sea cucumbers, abalone, and other animal seafood are rich in MSPs and other functional components, and they can also alleviate the metabolic syndrome induced by a high-fat diet [62,94]. Eating more of these seafoods seems to be beneficial for health, but too simple a diet is harmful for metabolic syndrome. Dietary intervention through intake of MSPs is a good way to prevent and alleviate metabolic syndrome and can provide reference for the development of functional foods in the future.

## 8. Conclusions and Future Perspectives

Metabolic syndrome is a universal health problem facing the world, and its incidence is increasing rapidly. It aggravates metabolic abnormalities and is associated with a marked increase in type 2 diabetes and early onset of cardiovascular and cerebrovascular risk. Interventions for metabolic syndrome are mainly lifestyle changes and necessary medication. MSPs are natural compounds in food, with a variety of biological activities, especially in the prevention and treatment of metabolic syndrome. Compared with traditional drugs, MSPs are safe and have low side effects, and they have been shown to have positive effects on obesity, insulin resistance, dyslipidemia, and hypertension.

The mechanism of MSPs prevention or treatment of metabolic syndrome may be through its regulation of glucose metabolism and lipid metabolism, and these regulation effects may be derived from the digestive products of MSPs in the gut and its effect on gut microbiota. MSPs can reduce the ratio of Firmicutes to Bacteroidetes, thus reducing the risk of obesity. In addition, it can increase the abundance of *Akkermansia* and other beneficial bacteria. After MSPs are degraded in the gut, SCFAs are generated; thus, the content of SCFAs and the abundance of SCFA-producing bacteria increase. SCFAs further affect glucose and lipid metabolism through complex mechanisms. It is generally believed that MSPs have higher activity due to their high sulfate content and sulphation patterns, and they are essential to the activity of MSPs. Intestinal sulfate-reducing bacteria may play an important role in the functioning of MSPs, but the effect and mechanism are still unclear.

MSPs have the potential to prevent and treat metabolic syndrome, which has a high value for drug development. At present, some clinical trials targeting fucoidan have been carried out, but other MSPs still need more animal and clinical trials to verify their function and safety. In addition, it is still not clear what role MSPs play in anti-metabolic syndrome and the mechanisms necessary for development of MSPs drugs. At present, there are still many difficulties in developing MSPs as drugs, but most MSPs can be added into the daily diet as functional food for dietary intervention to prevent and alleviate metabolic syndrome. In the future, it will be of great value to develop more MSPs formula foods for special medical use with clear function.

## Figures and Tables

**Table 1:** The effects of MSPs on the metabolic syndrome.

MSPs	Compounds/Extract	Source	Molecular Weight	Model	Positive/Negative Controls	Administration	Effects	References
sulphated polysaccharide	extract	*Cymodocea nodosa*	-	male Wistar rats with high fat diet	BC: normal rats were fed a standard laboratory diet PC: the rats received high-fat diet and treated with Orlistat NC: rats were fed a high-fat diet	gastric gavages route (200 mg/kg of body weight/daily) for 42 days	body weight ↓ lipase activity ↓ superoxide dismutase activity ↑ catalase activity ↑ glutathione peroxidase activity ↑ Thiobarbituric acid reactive substances levels ↓	[21]
fucoidan	extract	*Ascophyllum nodosum*	-	the human gut microbiota in vitro	PC: fermentation system with fructooligosaccharide BC: fermentation system without any addition	1 mL of the fecal inoculum was mixed with 9 mL of basal nutrient medium containing 90 mg of fucoidan	modulated the composition of the gut microbiota the relative abundance of Bacteroidetes and Firmicutes ↑ the total SCFA content ↑	[22]
sulfated polysaccharide	compound	abalone gonad	9 kDa	male BALB/c mice with high fat diet	BC: fed with a normal diet NC: fed with high fat diet	orally or gavage administrated with 2 mg the polysaccharide as drinking water (5 mL/day)	weight gain ↓ fat accumulation ↓ lipid droplets ↓ the level of Bacteroidetes ↑ the level of Firmicutes ↓ SCFAs production ↑ the expression of GPR43, aP2, UCP2, and LPL ↓	[23]
sulfated polysaccharide corresponding to fucosylated chondroitin sulfate and fucoidan	compound	*Stichopus japonicus*	179.4 kDa and >670 kDa,	diet-induced obese BALB/c mice	BC: fed a standard maintenance diet MC: fed with high fat diet	oral dosage of 300 mg/kg·d the sulfated polysaccharide	body weight ↓ fat and liver hypertrophy ↓ insulin resistance ↓ serum lipid ↓ inflammatory cytokine levels ↓ gut tissue index ↑ LPS-binding protein ↓ SCFA ↑ microbiota diversity ↑ probiotic *Akkermansia* ↑ endotoxin-bearing proteobacteria ↑	[24]
fucoidan	extract	*Sargassum fusiforme*	-	male ICR mice with high fat diet	BC: fed a standard maintenance diet MC: fed with high fat diet	physiological saline with fucoidan at 200 mg/kg intragastrically once per day for 6 weeks	blood glucose and insulin resistance index ↓ the levels of MDA and 4-HNE-modified protein ↓ GSH/GSSG ratio ↑ antioxidant enzymes ↑ activated Nrf2 signaling the abundance and diversity of gut microbiota ↑ improve intestinal integrity and inflammation	[25]
fucoidan	extract	*Fucus vesiculosus*	-	obese, with no history of diabetes, and subjects of ages between 18 and 65 years	BC: taken placebo capsules twice daily	active fucoidan 500 mg twice daily for 90 days	no marked effect on insulin resistance in obese, nondiabetic subjects	[26]
Sulfated fucan-dominated polysaccharide fraction fucosylated chondroitin sulfate-dominated polysaccharide fraction	extract	*Thelenota ananas* *Cucumaria frondosa*	-	high fat diet and streptozotocin induced type 2 diabetes male Sprague Dawley rat model	BC: fed with a normal diet MC: fed with high fat diet PC: fed with high fat diet and administration with 250 mg/kg/d metformin	200 or 400 mg/kg/d by daily oral gavage for 8 weeks	ameliorate hyperglycemia restore hypertriglyceridemia and hypercholesterolemia inflammatory status and oxidative stress ↓ protect against liver injury improve insulin resistance accumulation of hepatic glycogen ↑ activate IRS/PI3K/AKT signaling regulate GSK-3β gene expression	[27]
fucoidan	extract	brown seaweeds	-	spontaneously hyperlipidemic mice with high fat diet	BC: fed with a normal diet NC: fed with high fat diet	fed with 1% or 5% fucoidan	tissue weight (liver and white adipose tissue) ↓ blood lipid ↓ total cholesterol ↓ triglyceride ↓ non-high-density lipoprotein cholesterol ↓ glucose levels ↓ plasma lipoprotein lipase activity ↑ HDL-C levels ↑ hepatic steatosis levels (liver size, TC and TG levels, and lipid peroxidation) ↓ white adipose tissue LPL activity ↑	[28]
sulphated polysaccharide	extract	*Sargassum vulgare*	-	obese male Wistar rats	BC: fed a standard laboratory diet MC: fed a high-fat diet PC: fed a high-fat diet and treated with Orlistat (30 mg/kg, body weight/daily)	gastric gavages route (200 mg/kg of bodyweight/daily) for 6 weeks	body weight ↓ lipase activity ↓ antioxidant enzymes activities ↑ lipid peroxidation ↓ protect liver-kidney functions the levels of toxicity parameters in blood ↓	[29]
4-O-sulfation pattern fucodian	compound	*Pearsonoturia graeffei*	320 kDa	Sprague–Dawley rats with high fat diet	BC: fed a standard laboratory diet MC: fed a high-fat diet PC: fed a high-fat diet and treated with simvastatin (5 mg/kg)	oral administration (40 mg/kg) for 28 days by gavage	body weight regulate lipid disorder improve liver function adiponectin level ↑	[30]
ulvan	compound	*Ulva pertusa*	143 kDa	the model of hyperlipidemic Kunming mice	BC: fed a standard laboratory diet MC: fed a high-fat diet PC: fed a high-fat diet and treated with colestyramine (500 mg/kg)	oral administration (250 mg/kg body weight) for 28 days	antioxidant activity ↑ malondialdehyde ↓ superoxide dismutase ↑ catalase ↑	[31]
fucoidan	compound	*Laminaria japonica*	7 kDa	Goto-Kakizaki type 2 diabetic rats	BC: Wistar control rats MC: Goto-Kakizaki diabetic rats PC: Goto-Kakizaki diabetic rats treated with probucol (100 mg/kg)	fucoidan (50, 100, or 200 mg/kg/day) were given by intragastric administration for 12 weeks	basal hypertension ↓ ameliorate impairment of endothelium-dependent relaxation in the aorta, as well as mesenteric and paw arteries in diabetic rats eNOS phosphorylation at Ser1177 ↑ eNOS expression ↑ NO production ↑	[32]
fucoidan	extract	*Laminaria japonica*	6.5 kDa	instreptozotocin-induced type 1 diabetic rats	BC: streptozotocin-induced diabetic rats PC: streptozotocin-induced diabetic rats treated with probucol (100 mg/kg/day)	intragastric administration of fucoidan (50 or 100 mg/kg/day) for 12 weeks	body weight-loss ↑ hypertension ↓ hyperlipidemia ↓ serum level of total cholesterol, triglyceride, and low-density lipoprotein cholesterol ↓ ameliorate STZ-elicited hyper-responsiveness and oxidative stress in aortic smooth muscles superoxide level ↓ glutathione content and higher superoxide dismutase activity ↑ prevent cyclooxygenase-2 stimulation thromboxane synthase ↑ 6-keto-PGF1α ↓	[33]
fucoidan	compound	*Undaria pinnatifida*	54 kDa	eNOS inhibition-induced hypertensive Sprague-Dawley rats	BC: the normotensive group placed on a basal diet MC: L-NAME-induced hypertension rats PC: L-NAME-induced hypertension rats treated with nifedipine (5 mg/kg)	administered at 20 mg/kg/day or 100 mg/kg/day by daily gavage for four weeks	hypertension ↓ NO production ↑ activate eNOS and Akt phosphorylation protect against vascular structure damage enhance endothelium-independent vascular function and inhibit abnormal proliferation of smooth muscle cells vascular inflammation and oxidative stress ↓	[34]
carrageenans	extract	*Sarconema filiforme*	-	high-carbohydrate, high-fat diet-fed male Wistar rats	BC: fed either corn starch or high carbohydrate, high-fat diets	supplemented with 5% *S. filiforme* power for the last 8 weeks	body weight ↓ abdominal and liver fat ↓ systolic blood pressure ↓ plasma total cholesterol concentrations ↓ plasma activities of alanine transaminase and aspartate transaminase ↓ modulate gut microbiota without changing the Firmicutes to Bacteroidetes ratio infiltration of inflammatory cells into organs ↓	[35]

Abbreviations: SCFA, short chain fatty acid; ↑, increase; ↓, decrease; ICR, institute of cancer research; GPR43, G protein-coupled receptor 43; aP2, adipocyte lipid-binding protein 2; UCP2, uncoupling protein-2; LPL, lipoprotein lipase; LPS, endotoxin; MDA, malondialdehyde; 4-HNE, 4-hydroxynonenal; GSH/GSSG, the ratio of reduced glutathione to oxidized glutathione; Nrf2, NF-E2-related factor 2; IRS, insulin receptor substrate; PI3K, phosphatidylinositol 3-kinase; AKT, protein kinase B; GSK-3β, glycogen synthase kinase 3β; HDL-C, high-density lipoprotein cholesterol; TC, total cholesterol; TG, triglyceride; eNOS, endothelial nitric oxide synthase; NO, nitric oxide; STZ, streptozotocin; 6-keto-PGF1α, a stable metabolic product of prostaglandin I_2_; Akt, protein kinase B; L-NAME, Nω-nitro-L-arginine methyl ester; -, not detected; BC, black control; PC, positive control; NC, negative control; MC, model control.

**Table 2:** The regulation of MSPs on gut microbiota.

MSPs	Source	Compounds /Extract	Molecular Weight	Model	Positive/Negative Controls	Administration	Effects	References
Chondroitin sulfate	-	compounds	24 kDa and 130 kDa	Kunming mice	NC1: male mice oral administrated with normal saline NC2: female mice oral administrated with normal saline	administration of 150 mg/kg by gavage once a day for 6 weeks	sex-dependent effect on gut microbiota	[61]
Fucosylated chondroitin sulfate	*Isostichopus badionotus*	compounds	10.9 kDa	C57BL/6 mice with high-fat and high sucrose diet	BC: mice were fed on regular chow NC: mice were fed on a high-fat and high sucrose diet	administration of 20 or 40 mg/kg/day by metallic gavage needle for 6 weeks	alleviate obesity, hyperlipidemia, hyperglycemia, inflammation, liver steatosis, and adipocyte hypertrophy ratio of Firmicutes to Bacteroidetes ↓ Lachnospiraceae and Allobaculum ↓ Porphyromonadaceae, Barnesiella, and Bacteroides ↑	[62]
Fucosylated chondroitin sulfate	*S. chloronotus*	compounds	63.2 kDa	in vitro fermentation with fecal slurry	PC: fructo-oligosaccharide were dissolved in culture medium at 10 mg/mL NC: blank culture medium	-	absolute abundance of microbiota ↑ Megamonas, Bacteroides, Fusobacterium, Parabacteroides, Prevotella, Faecalibacterium ↑ short-chain fatty acids ↑	[63]
fucoidan	*Ascophyllum nodosum* and *Laminaria japonica*	extract	-	specific pathogen-free male C57BL/6 mice	NC: oral administration of normal saline	100 mg/kg/day by gavage for 6 weeks	Lactobacillus and Ruminococcaceae ↑ a more balanced composition of gut microbiota serum lipopolysaccharide-binding protein levels ↓	[64]
fucoidan	*Sargassum fusiforme*	extract	205.8 kDa	Pathogen-free male ICR mice with high-fat diet and streptozotocin induced mouse model of type 2 diabetes	BC: healthy mice fed with a normal chow diet NC: diabetic mice administered normal saline	100 mg/kg/day by gavage for 4 weeks	blood glucose ↓ diet and water intake ↓ hyperlipidemia ↓ glucose tolerance ↑ epididymal fat deposition ↓ pathological changes in heart and liver tissues ↓ oxidative stress ↓ Bacteroides, Faecalibacterium and Blautia ↑ levels of (R)-carnitine and choline in the colon ↑	[65]
fucoidan	*Sargassum fusiforme*	extract	205.8 kDa	male ICR mice with streptozotocin-induced hyperglycemia	BC: normal mice treated with distilled water NC: diabetic mice with distilled water	100 mg/kg/day by gavage for 6 weeks	blood glucose ↓ diet and water intake ↓ the pathological change in the heart and liver ↓ improve the liver function suppress oxidative stress decrease the relative abundances of the diabetes-related intestinal bacteria	[66]
Carrageenan oligosaccharides	Lubao Biochemistry, China	compounds	-	in vitro fermentation with fecal slurry	PC: the medium contained 10 μg LPS NC: basal nutrient medium	-	pro-inflammatory bacteria Prevotella ↑ anti-inflammatory bacteria Bacteroides and Parabacteroides ↓ KO3 (larger degrees of polymerization): Bifidobacterium and Lactobacillius ↑ and SCFAs ↑ KO6 (smaller degrees of polymerization): Prevotellaceae ↑ and SCFAs ↓	[67]
Carrageenan oligosaccharides	Lubao Biochemistry, China	compounds	-	HT29 cells	BC: the cells were treated with fresh DMEM medium for 24 h. PC: the cells were treated with culture medium containing LPS (1 μg/mL) for 24 h	Treatment at 50, 100, and 200 μL/mL for 24 h.	KO6: IL-1β, TNF-α, SIgA and mucin2 ↑	[67]
carrageenan	*Kappaphycus alvareziiwere*	extract	365 kDa	male SPF C57BL/6J mice with high fat diet	BC: low fat diet + normal water NC: high fat diet + normal water	administration of 0.5% or 5% carrageenan in drinking water for 6 weeks	disease activity index ↑ myeloperoxidase activity ↑ mRNA expression of Toll-like receptor 4 in colon ↑ inflammatory-causing bacteria Alistipes finegoldii and Bacteroides acidifaciens ↑	[68]
laminaran	Carbosynth, UK	compounds	-	in vitro fermentation with fecal slurry	NC: basal culture medium	-	Bifidobacteria and Bacteroides ↑ acetate and propionate ↑	[69]
Ulvan	Carbosynth, UK	compounds	-	in vitro fermentation with fecal slurry	NC: basal culture medium	-	Bifidobacteria and Lactobacillus ↑ lactate and acetate ↑	[69]
porphyran	Carbosynth, UK		-	in vitro fermentation with fecal slurry	NC: basal culture medium	-	little prebiotic effect	[69]

Abbreviations: ↑, increase; ↓, decrease; -, not detected; ICR, institute of cancer research; SCFAs, short chain fatty acids; IL-1β, interleukin-1β; TNF-α, tumor necrosis factor-α; SIgA, secretory immunoglobulin A; SPF, specific pathogen free; BC, black control; PC, positive control; NC, negative control.

**Table 3:** Clinical data of MSPs in the treatment of metabolic syndrome.

MSPs	The Number of Patients	Disease	Dosing Regimen	Timing	Results	References
fucoidan	24	healthy participants	1 mg fucoxanthin (6 participants), 400 mg fucoidan (9 participants), and both (9 participants) administered to volunteers	5 weeks	significantly shortened lysis time of the thrombu H_2_O_2_ ↑ the secretion of prostacyclin ↑	[91]
	25	obese or overweight	13 patients received an oral dose of 500 mg of fucoidan once daily before breakfast and 12 patients received placebo	3 months	Diastolic blood pressure ↓ low-density lipoprotein cholesterol ↓ insulin ↑ HOMA β-cell ↑ HOMA insulin resistance ↑	[92]
	20	obese, with no history of diabetes	active fucoidan 500 mg or placebo capsules twice daily	90 days	no differences the mean change in HOMA scores was 0 for the placebo and −0.1 for the active groups	[25]
	42	non-alcoholic fatty liver disease	275 mg fucoidan and 275 mg fucoxanthin twice per day in the treatment group, or placebo (550 mg/capsule cellulose powder) in the control group	24 weeks	alanine aminotransferase ↓ aspartate aminotransferase ↓ total cholesterol ↓ triglyceride ↓ fasting blood glucose ↓ hemoglobin a1c ↓ the scores of controlled attenuation parameter ↓ adiponectin and leptin expression ↑	[93]
carrageenan	13	prediabetes	8 patients were provided all meals with no carrageenan. 5 patients received a similar diet with carrageenan (total estimated to be 250 mg/day)	12 weeks	no significant declines in Hemoglobin a1c or HOMA-IR C-peptide ↑ phospho-serine-insulin receptor substrate 1 ↑ phospho-serine-protein kinase 1 ↓ mononuclear cell arylsulfatase B ↓	[94]

Abbreviations: ↑, increase; ↓, decrease; HOMA: homeostasis model assessment.

## Data Availability

The data presented in this study are available on request from the corresponding author.
